# Evaluation of Praziquantel effectiveness in treating Nile tilapia clinostomid infections and its relationships to fish health and water quality: By

**DOI:** 10.1186/s12917-024-04279-2

**Published:** 2024-10-05

**Authors:** Olfat A. Mahdy, Marwa M. Attia, Iman B Shaheed, Mohamed Abdelsalam, Mamdouh Y. Elgendy, Mai A. Salem

**Affiliations:** 1 Department of Parasitology, Faculty of Veterinary Medicine, Cairo University, Giza, 12211 Egypt; 2 Department of Pathology, Faculty of Veterinary Medicine, Cairo University, Giza, Egypt; 3 Department of Aquatic Animal Medicine and Management, Faculty of Veterinary Medicine, Cairo University, Giza, Egypt; 4 Department of Hydrobiology, Veterinary Research Institute, National Research Centre, Dokki, 12622 Cairo Egypt

**Keywords:** Clinostomid, *Clinostomum*, Molecular characterization, Water analysis, Blood parameters, PZQ, Freshwater fishes

## Abstract

This study aimed to conduct a multidisciplinary investigation integrating detailed morphology, molecular characterization, water parameters, histopathology alteration, and the trials of treatment of *Clinostomum* spp. In this study, 300 Nile tilapia (*Oreochromis niloticus*) were collected from the farmed and wild Nile River at Al Bahr Al Aazam, Giza Governorate to assess Clinostomid infection prevalence. Fish and water samples were collected from private fish farms, and water drains at Dakahlia, and Giza, Egypt. Analysis of the water revealed inadequate water quality, particularly in the fish farms. Snails and piscivorous birds were abundant at fish collection sites. The recovered Clinostomid MCs morphological characteristics and COI gene sequence analysis identified them as *Clinostomum complanatum*, *C. phalacrocoracis*, and *Euclinostomum heterostomum*. Clinostomid MCs disturbed the fish’s hematological and biochemical blood parameters. Bath treatment of parasitized fish with praziquantel (2 mg/L for 24 h) revealed a significant reduction in the number of vital MCs vs. infected fish (non-treated). Praziquantel (PZQ) is an effective and safe therapy for controlling Clinostomid infections affecting farmed Nile tilapia. The current findings indicate a link between poor environmental conditions and *Clinostomum* infections in tilapia. The study highlights the impacts of Clinostomid MCs on fish health and recommends bath treatment with PZQ as an efficient control method for these dangerous parasites to protect human and fish health.

## Introduction

Parasites negatively impact fish health through physical injury, competition for food, and disruption of their metabolic, physiological, and immune processes [1, 2]. The major threat to the global spread of parasitic disorders caused by the causative agent helminthes is the large number of infected hosts, including humans [3]. In recent years, there has been a growing public awareness concerning fish borne zoonotic trematodes [4, 5]. Clinostomiasis is a dangerous fish parasitic infection caused by metacercarial infection of the genus *Clinostomum* [6, 7]. Recent studies on the impact of Clinostomiasis on fish hosts provide crucial data to control this recently identified fish-borne zoonotic disease currently threatening aquaculture, wildlife, and human health [8]. Furthermore, fish-eating birds, reptiles, and mammals are the definitive hosts of these parasites [9–11]. The Clinostomid worms; *Clinostomum complanatum*, *C. phalacrocoracis*, and *Euclinostomum heterostomum* are among the most dangerous Clinostomid species affecting fish [12, 13]. Fish infection with Clinostomid MC is critical to commercial fish producers because their presence reduces fish marketability and consumers appeal [14–17]. Moreover, Praziquantel (PZQ) is a synthetic drug that was discovered by Bayer in the 1970s [18]. Remarkably, PZQ is effective against a broad range of cestodes (tapeworms) and trematodes (flukes) and is a mainstay of anti-platyhelminth parasite therapy in both human and veterinary medicine [19]. The anthelmintic PZQ can effectively treat a range of flatworm parasites; *Hymenolepis nana* by [20] and in a variety of fish species and has potential for broader application than its current use in the global aquaculture industry [21]. Fish parasites may be helpful markers of pollution. Aquatic animals are known to be impacted by a variety of contaminants, such as heavy metals and domestic sewage. The majority of fish parasites are thought to do little to no harm in their natural environments, while many of the latter are parasitized [22].

Therefore, the present objectives were investigated the prevalence and intensity of Clinostomid metacercariae infections in farmed and wild fish, and characterize morphological and molecular features of *Clinostomum* and *Euclinostomum* to distinguish between species. Moreover, we assessed water quality and environmental factors predisposing fish to Clinostomid infections, and evaluate changes in blood biochemistry of infected fish. Additionally, we explored the efficacy of praziquantel in controlling Clinostomiasis and histopathological alternative in host tissues and organs resulting from *Clinostomum* infections.

## Materials and methods

### Study area and fish samples

The study was conducted in August 2022, involving the collection of 300 Nile tilapia (*Oreochromis niloticus*) 150 wild and 150 farmed to investigate clinostomid parasitic infections. Farmed tilapia, with a mean body weight of 171.0 ± 1.0 g, were sourced from private fish farms in Dakhalia and Giza, Egypt. Observations at these farms included stunted growth, lethargy in fish, grazing by sheep and cattle near ponds, and an overgrowth of phytoplankton. Wild tilapia, with a mean body weight of 144.5 ± 2.5 g, were collected from water drains near fish farms, where fishermen noticed abnormal yellow grubs in the buccal cavities and on the skin. Both sites were heavily populated with snails and piscivorous birds, such as egrets and herons. Fish were collected by fishermen, transported alive to the parasitology laboratory at Cairo University, and maintained in aquaria with dechlorinated tap water under proper aeration for further examination.

### Water analysis

Water samples were obtained from the investigated fish farms and water drains and stored in plastic containers for further analysis [23]. The physicochemical characteristics of water were determined in the obtained samples following the methods described by [24]. Total and fecal coliform counts were enumerated in water samples as the most probable number (MPN/100 mL water) by the multiple-tube fermentation method [25].

### Clinical examination

Fish samples were clinically examined, and abnormal signs were recorded. The external and internal gross lesions were noticed as criteria [7, 26].

### Parasitological examination

Fish were euthanized by overdosing them with anesthetics MS-222 (Sigma), sacrificed, and carefully examined to detect parasitic infections following standard protocols described by [26]. The Clinostomid cysts were investigated in the examined fish specimens following methods described by [7]. External body surfaces, gills, fins, eyes, gill chambers, buccal cavities, muscles, kidneys, brain, internal organs, and every part of fish were carefully examined macroscopically and microscopically for metacercarial cysts. The cyst was fixed between two glass slides in 70% ethanol. The parasites were stained with acetic acid alum carmine, dehydrated with graded concentrations of alcohol, cleared in clove oil, mounted in Canada balsam, and examined by light microscopy [8]. The measurements of EMC were recorded using a light microscope (Olympus CX41 microscope, Japan) and expressed as mean ± S.E. The fish parasite prevalence (P) and mean intensity (MI) were calculated according to [9]. Parasites were identified based on international keys for the families Clinostomidae by [7].

### Molecular identification

Fifteen specimens of MCs of each species were washed and cleaned with sterile saline; then, every five specimens were preserved at -20 °C in an Eppendorf tube. Genomic DNA from cyst was extracted using the QIA amp DNA Mini kit (Qiagen, Germany) according to the tissue protocol provided by the manufacturer. The concentration and quality of extracted DNA were determined using the Nanodrop2000 spectrophotometer (NP80, Nanophotometer, Implen, Germany).PCR was performed to amplify the cytochrome oxidase subunit 1 (COI) gene using the specific degenerate primer pairs; MplatCOX1dF: (′5-TGT AAA ACG ACG GCC AGT TTW CIT TRG ATC ATA AG-3′) and MplatCOX1dR: (′5-CAG GAA ACA GCT ATG ACT GAA AYA AYA IIG GAT CIC CAC C-3′) [27, 28], . PCR amplifications were performed using Emerald Amp Max PCR Master Mix (Takara, Japan) in a 25 ul reaction volume. The thermal cycler was adjusted [4] with a few alterations. The PCR process started at 94 °C/ 2 min (initial denaturation) and succeeded by 35 cycles of (denaturation-94 °C/30 s), (annealing-50 °C/30 s), and (extension-72 °C/ 1 min), and then ended at 72 °C/ 10 min (final extension). Purification of amplicons was carried out with a QIA quick PCR purification kit (Qiagen, USA). For bidirectional sequencing, PCR products of the COI gene were submitted with the same primer pairs to Macrogen Inc. (Macrogen, Seol, South Korea). The sequencing process was carried out using the Big Dye terminator cycle sequencing kit, and electrophoresis was accomplished with the 3730XL sequencer model (Applied Biosystems™, USA). The raw data of sequences were edited and assembled using the BioEdit software. The final sequences were submitted to GenBank to issue the corresponding accession numbers. The assembled sequences were then compared against other sequences using the BLAST program of NCBI.

The phylogenetic tree was constructed to compare the three sequences of the COI gene against 43 various sequences verified for *C. complanatum*,* C. phalacrocoracis*,* C. sinensis*, *C. chabaudi*,* C. tilapiae*,* C. philippinense*,* C. brieni*,* C. cutaneum*,* C. ukolii*,* C. detruncatum*,* C. attenuatum*,* C. album*,* C. marginatum*,* C. tataxumui*,* E. heterostomum*, and *Diplostomum mergi*. The multiple sequence alignment was performed using the CLUSTAL W program. The homologous similarities of the six sequences were determined using the neighbor-joining methods of MEGA 11 with 95% cutoff partial deletion principal and 1000 bootstrap values.

### Hematological analysis

Hematological analysis was performed only on the farmed Nile tilapia samples following the methods [28]. We could not collect blood samples from wild tilapia because they died shortly after arrival at the lab. Blood samples were collected from all farmed fish samples before performing the parasitological examination, and blood samples were coded. Blood samples were collected from the caudal vein of fish after anesthetizing them by MS-222 (Sigma) and categorized into two groups (infected and non-infected). Counts of red blood cells (RBCs) and white blood cells (WBCs) were performed using a Neubauer hemocytometer [29]. The following blood biochemical parameters were determined: glucose, uric acid, cholesterol, creatinine, total protein, albumen, globulin, Alkaline phosphatase (ALP), alanine aminotransferase (ALT), aspartate aminotransferase (AST). The lysozyme activity was measured in fish sera as [30], by the turbidimetric assay using a suspension of 0.2 mg/ml *Micrococcus lysodeikticus* (Sigma-Aldrich).

### Scanning electron microscopy (SEM)

Parasite specimens were prepared for SEM following the methods described by [12]. Parasites were washed in PBS, fixed with glutaraldehyde (2.5%), and dehydrated with graded ethanol series. Specimens were dried in a carbon dioxide critical point drier (Autosamdri-815, Germany) and coated with 20-nm gold particles in a sputter coater (Spi-Module Sputter Coater, UK). The prepared specimens were examined by a scanning electron microscope (JSM 5200 electron probe microanalyzer, JEOL, Japan) at magnifications ranging from 35X to 500X [8].

### Treatment trials of Praziquantel (PZQ)

Treatment trials were performed to test the efficacy of PZQ (Biltricide ^®^, Bayer, Germany) against the *Clinostomum* grubs. Nile tilapia (*n* = 135), naturally infected with *Clinostomum* grubs, averaging 45–75 g, were collected from the same investigated farms and transferred to the laboratory. Fish exhibiting signs suggestive of *Clinostomum* infections, like stunted growth or the presence of grubs, were selected. Fish were kept in glass aquaria (70 L) with aeration at 25 ± 1 °C. After the acclimatization of the fish, they were divided into 9 main groups (control and 8 treatment groups), each with 15 fish (5 × three replicates). Before starting the treatment trials, random fish samples (5) were collected from each tank and examined for *Clinostomum* grubs to ensure their presence in fish. The remaining fish (*n* = 10) in each aquarium were treated with different concentrations of PZQ according to [18, 20]. The drug was dissolved with alcohol and added in different concentrations to the aquaria. Different treatment protocols were applied with low, high, short and prolonged exposure times are display in Table (1). After ending drug-exposure time, all fish were transferred to fresh, clean water and kept under observation. Fish were examined for *Clinostomum* after 14 days of the treatments. Treatment efficacy was based on the percentage of dead metacercarial cysts to the total metacercarial cysts.

**Table 1:** Efficacy of praziquantel in treating clinstomid infections in tilapia

	Praziquantel Dose (mg/L)	Exposure time (hour)	Trials number
Control fish a	0	-	-
c	2	24	1
d	3	24	1
e	0.5	24	2
f	2	24	2
g	3	24	2
h	4	4	1
i	8	4	1

### Histopathology

Samples were collected from each infected muscle and kidney of a different fish species (Nile tilapia), fixed in 10% buffered formalin, and processed [31]. Sections were deparaffinized and stained with hematoxylin and eosin for histological examination using light microscopy. Muscles and kidneys were examined and photographed using an Olympus CX41 microscope [7].

### Statistical analysis

All data are described as means ± standard error (SE). Significant differences in the detected values between the control fish and other experimental groups were determined using the one-way ANOVA test [7]. The SPSS (version 17.0 for Windows) software (SPSS Inc.) at *p* < 0.05was used in all statistical analyses.

## Results

### Water analysis

The results of the water analysis are summarized in Table (2). The physicochemical analysis of water revealed unfavorable water quality measures at both sites. The water quality indicators were more deteriorated in the fish farm water than in water drains. Stressful values of with high level (1.58 ± 0.0) and low level of dissolved oxygen (4.1 ± 0.21) from farm water were detected. Results indicated the presence of coliforms in water referring to fecal contamination. High bacterial and coliform counts were discovered in the water, with the highest concentrations being noticed in tilapia farms. Additionally, high bacterial and coliform counts were found in the water, with the highest concentrations observed in the tilapia farms.

**Table 2:** Physicochemical analysis of water (mean ± SE)

Parameter	Tilapia fish farm	Water drains
Water temperature (^o^C)	29 ± 3	26 ± 2
Dissolved Oxygen (mg/L)	4.1 ± 0.21	5.17 ± 0.14
pH	7.83 ± 0.15	6.86 ± 0.10
TDS (mg/L)	899 ± 6.5	755 ± 3.00
Total alkalinity (mg/L)	197.61 ± 3.52	221 ± 2.30
Total hardness (mg/L)	218.43 ± 2.51	245.67 ± 1.44
Sulfate (mg/ L)	114 ± 2.74	129 ± 3.95
phosphates	0.21 ± 0.04	0.38 ± 0.02
Ammonia (mg/L)	1.58 ± 0.04	0.95 ± 0.02
NO2 (mg/L)	0.51 ± 0.03	0.37 ± 0.02
NO3 (mg/L)	0.91 ± 0.01	0.58 ± 0.03
Total colony count ×10^3^ CFU /mL	389.23 ± 2.06	296 ± 3.61
Total coliform ×10^3^ CFU /mL	279 ± 3.05	233.83 ± 1.64

(TDS) Total dissolved solid, (CFU) Colony forming unit

### Clinical examination

Fish showed stunted growth and emaciation. Farmed fish exhibited signs of respiratory distress, such as rapid opercular movement and swimming near the water’s surface. Gills appeared pale with excessive mucus secretions. The most prominent postmortem findings were the presence of characteristic yellow cysts and greyish cysts embedded in the buccal cavities and tissues of kidneys, respectively. Other postmortem lesions were extensive hemorrhagic patches surrounding the metacercarial attachment sites.

### Parasitological examination

**Fig. 1:**
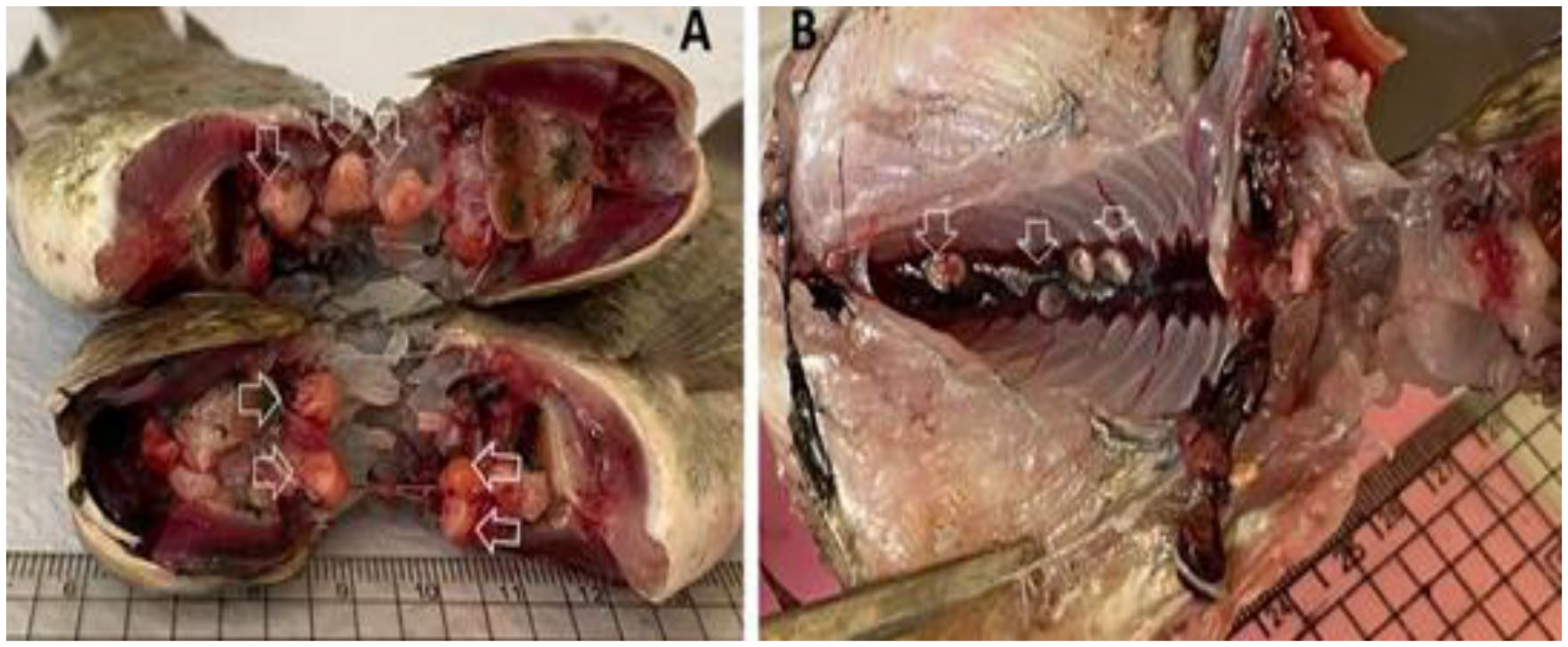
(**A**) Encysted MC of *Clinostomum* species; *C. phalacrocoracis* showed large-sized yellowish cysts infected buccal cavities. (**B**) EMC of *E. heterostomum* infected kidney showed a grayish spherical cyst infected kidney (arrows)

**Fig. 2:**
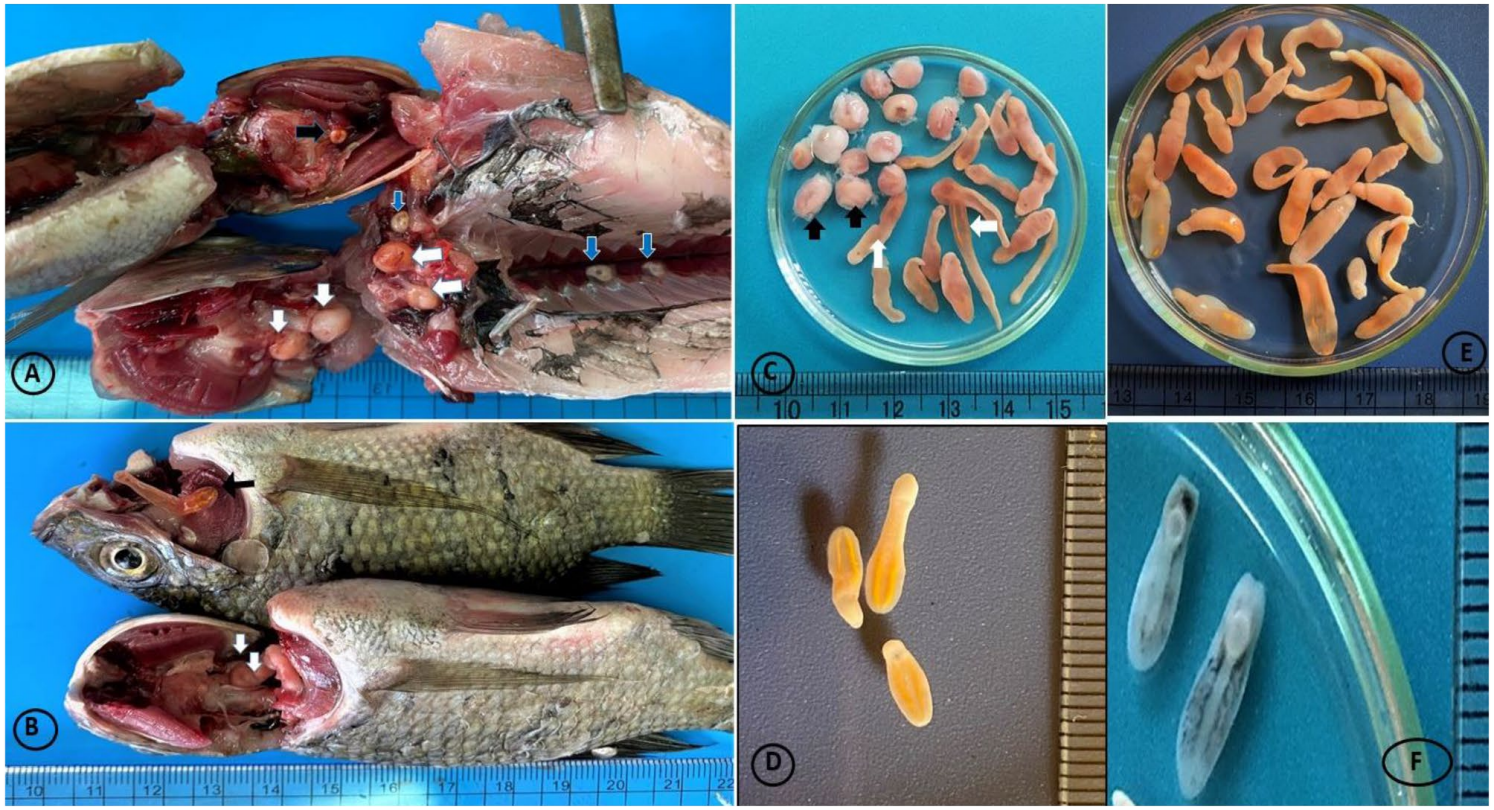
(**A**) Oreochromis *niloticus* Co infection of macroscopic EMC of yellow grub trematode, *C. complanatum* (black arrow); *C. phalacrocoracis*, (white arrow), *E. heterostomum* (blue arrows), EMC of *E. heterostomum* infected kidney showed greyish spherical cysts in the peritoneum covering the kidney (blue arrows). (**B**) *C. phalacrocoracis* MC (black arrow), EMC (white arrow). E) *C. phalacrocoracis* MC (D) Excysted MC of *C. complanatum*. F) Excysted MC of *E. heterostomum*

Investigated tilapia specimens were infected with Clinostomid cysts, which were identified at the species level based on the morphological characteristics of the meteacercariae and gene sequence analysis (Fig. 1 and 2).

### Prevalence and intensity

Table 3 summarizes the prevalence and pattern of intensity of MCs in investigated tilapia specimens. The prevalence of MCs was higher in farmed fish (32%) than in wild tilapia (24%). The intensity of parasites varied between investigated tilapia specimens. The mean parasite intensity per host was higher (6.2) in farmed fish than in wild tilapia (3.85). The highest number of CMCs collected from a single fish was 22 cysts.

**Table 3:** Prevalence, mean intensity of clinostomid MC in farmed and wild Nile tilapia

Fish species	No. exam fish	No. infected fish	Prevalence %	No. parasites	Mean intensity	Pattern of parasites intensity		Fish organs infected with clinostomid metacercarial species			
						Intensity	%	Infected organ	*C. phal*	*C. comp*	*E. het*
Wild tilapia	150	35	24	220	6.2	*	10 (6.6%)	skin	-	√	√
								Branchial cavity	√	√	-
						**	13 (8.6%)	Buccal cavity	√	√	√
						***	8 (5.3%)	kidneys	-	-	√
						****	1 (0.6%)	Abdominal cavity	-	√	√
Farmed tilapia	150	49	32	189	3.85	*	3 (2%)	skin	-	√	√
								Branchial cavity	√	√	-
						**	5 (3.3%)	Buccal cavity	√	√	√
						***	10 (6.6%)	kidneys	-	-	√
						****	6 (4%)	Abdominal cavity	-	√	√

Inten (Intensity), *Minimum (1–5 cyst), ** medium (5–10 cyst), *** High (10–15 cyst), **** Max (> 15 cyst), Mean for intensity of infection. *C. pha* (*C. phalacrocoracis*), *C. comp* (*C. complanatum*), *E. het* (*E. heterostomum*)

### Morphological description of Clinostomid MCs

Three MC species were noticed in the investigated tilapia specimens, *C. phalacrocoracis*, *C. complanatum*, and *E. heterostomum.* The *Clinostomum* cysts attached firmly to the infected fish tissues and were surrounded by thick capsules. Cysts varied in size; large with a diameter of (5.0–9.0 mm) and small (1.0–4.0 mm) cysts were noticed. The EMC of *Clinostomum* spp. noticed in the investigated fish specimens appeared as yellowish to white-yellow grape-like structures while *Euclinostomum* MCs were pea-shaped and greyish-white in color. The morphological description of Clinostomid worms was performed based on 10 samples of each parasitic species Table (3) and Fig. (1 A-C) & (2 A-B).

### *Clinostomum complanatum*

**Fig. 3:**
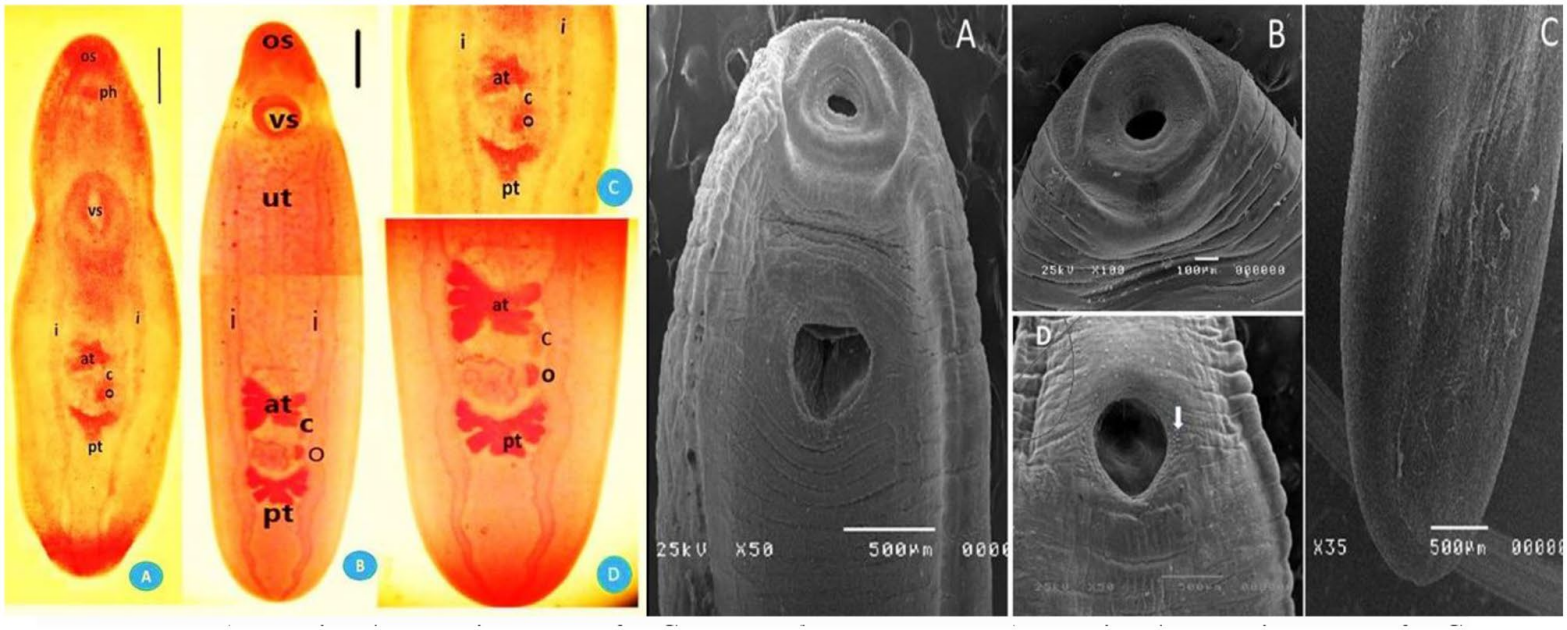
(**A**) Stained specimens of *C. complanatum.***B-C**) Stained specimens of *C. phalacrocoracis* worms; oral sucker (os), ventral sucker (vs.), intestinal ceca (i), uterine tube (u), anterior testis (at), posterior testis (pt), intestinal ceca (i)) scale bar 2 mm. **A-D**): SEM micrograph of *C. phalacrocoracis* MC. **A-B**) Ventral tegumental surface of the forebody exhibited; a collar-like ring and large ventral sucker. **D**) A large ventral sucker (vs.), ventral spongy fold provided with sensory papillae. **C**) Hind body exhibited smooth normal tegument without spines

The body of MC is flat and linguiform. The body is marginally narrower at the level of the ventral sucker region than at the level of the gonads. The oral collar is not developed. The mouth is in the center of the oral sucker, smaller than the ventral sucker. The intestinal ceca extend posteriorly and laterally to the genital organs. Testis is triangular. The anterior testes of C. *complanatum* MC, located at the end of the middle third of the body, are larger than their posterior counterparts, which exist in the middle third of the body. The genital pore is located at the midline of the body. The ovary is smaller than the cirrus sac and exists between two testes (Fig. 3A, C).

### *Clinostomum phalacrocoracis*

The MC body was stout and slightly wider in its gonadic region. The oral sucker was smaller than the oral collar. The pharynx is evident, and the intestine immediately divides behind it. The intestinal caeca extended to the ventral sucker. Testes are arranged in tandem in the posterior third of the body. The anterior testis is blunt-ended, fan-shaped, and consists of several lobes (3–7) subdivided into sub-lobes. The posterior testis presents in the body’s posterior third and comprises three subdivided lobes. The ovary is round and irregular and resides in between testicles. The uterus extends from the ventral sucker to the anterior testicle of the parasite (Fig. 3B,D). The ultrastructure observation of worm using SEM is shown in (Fig. 3A–D). Worms appeared to have distinct smooth transverse annulations and ridges. The oral sucker consisted of two distinct collar-like rings in a semicircular and fat shape, which was provided with sensory papillae. A large ventral sucker was closed and located near the oral sucker. A distinct ventral fold with a sponge-like character surrounded the ventral sucker, and dome-like papillae were present around the fold margins. The worms had a distinct, marked smooth and normal tegument structure at the hind body.

### *Euclinostomum heterostomum*

**Fig. 4:**
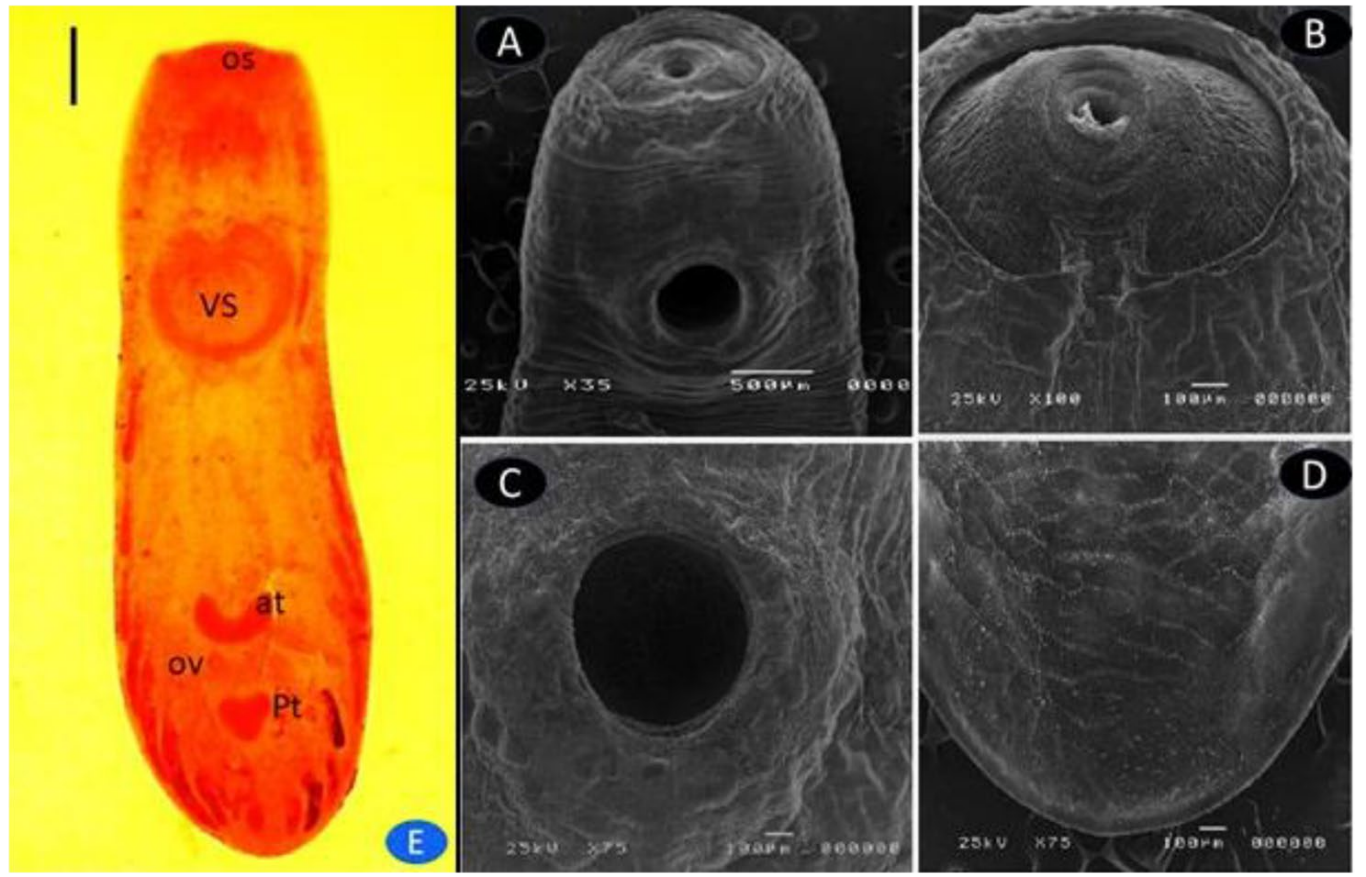
E) A Stained *E. heterostomum* worms; oral sucker (os), ventral sucker (vs.), intestinal ceca (i), uterine tube (u), anterior testis (at), posterior testis (pt), intestinal ceca (i)) scale bar 2 mm. A-D) SEM of *E. heterostomum* on the ventral tegument exposed to

**Fig. 5:**
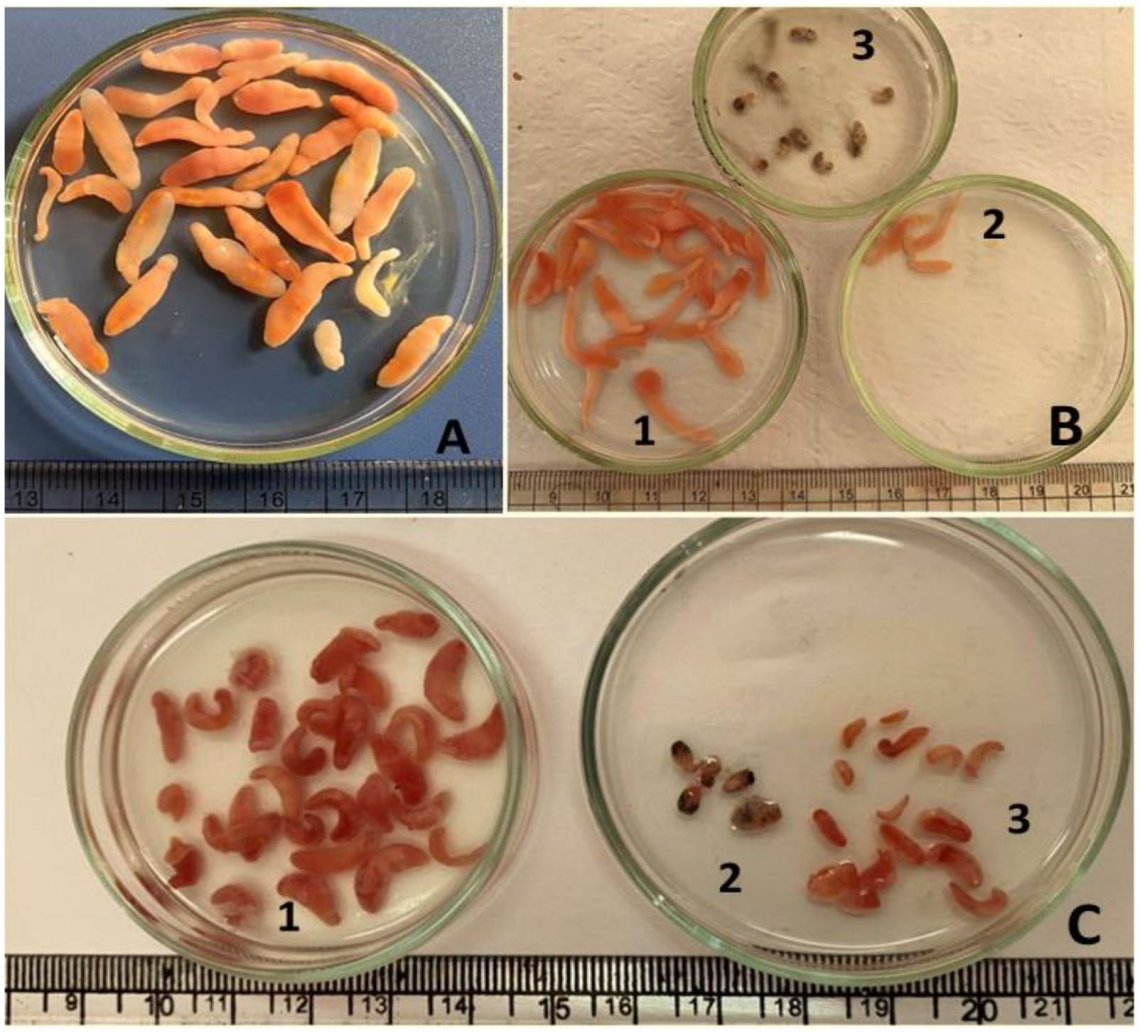
Untreated control group of MCS of *Clinostomid* species; A-B1) *C. phalacrocoracis* showed longed yellowish MC. B2) MC of *C. complanatum* showed short yellowish MC. B3) ExMC of *E. heterostomum* MC. C) Treated group of MCs of *Clinostomid* species;1) *C. phalacrocoracis* showed dark color and shrink MC. 3) MC of *E. heterostomum* showed a dark color and shrined worms. 3) *C. complanatum* showed a dark color and shrunk worms

The *E. heterostomum* EMC was spherical and pale to dark yellow. The *E. heterostomum* cyst wall was very dense (Figs. 1 and 2). The body of the *E. heterostomum* metacercaria has the shape of a broad slipper with a restricted ventral sucker region and a broad gonadal region. The oral sucker of *E. heterostomum* is smaller than its ventral sucker. The small intestine divides Just posterior to the pharynx. The Intestinal ceca extend laterally up to the ventral sucker and are enlarged in the pre-acetabular region. The major ceca form secondary numerous blind diverticulitis behind the ventral sucker. The excretory pore exists posteriorly at the end of the body. The testes are located in the middle and posterior thirds of the body in the intercaecal region. The anterior testis has a crescent to U-shaped configuration. The posterior testis is large, and its anterior border is concave. The genital pore opens on the margin of the anterior testis. The vitellaria are not obvious, and the tegument surface is spineless (Fig. 4A). In addition, SEM: the worm showed tegument of the oral sucker was smooth collar-like rings, covered by ridges (Fig. 4A-B). The ventral sucker exhibited was sponge-like with normal tegument margins (Fig. 4C). Body posteriorly showed normal tegument (Fig. 4C and 5).The wormbody was truncated anteriorly and broadly rounded posteriorly. Bodies were divided by a slight constriction into a narrower pre-acetabular and broader posterior parts (Fig. 4D).The morphometric characteristics of all recovered Clinostomid metacercarial species are displayed in Table (4).

**Table 4:** Morphometric measurements of collected Clinostomid encysted metacercaria (*n* = 10); Min-max (mean ± SE) (mm

Morphometry body parameters	*C. complanatum*	*C. phalacrocoracis*	*E. heterostomum*
EMC length	6.7–9.44 (5.15 ± 0.4)	12.5–19.4 (1.8 ± 0.4)	6.6–9. 8 (7.0 ± 0.5)
EMC width	1.5–2.8 (1.93 ± 1.9)	2.7–3.9 (2.9 ± 0.4)	2.4–3.9 (2.55 ± 0.5)
Oral collar	0.39–0. 80 (0.73 ± 0.50)	0.66–1.21 (1.0 0 ± 0.04)	0.44–1.3 (0.52 ± 0.12)
Oral sucker length	0.12–0.37 (0.24 ± 0.015)	0.35–0.50 (0.42 ± 0.02)	0.22–0.37 (0.29 ± 0.03)
Oral sucker width	0.3–0.5 (0.39 ± 0.02)	0.5–0.8 (0.6 ± 0.03)	0. 4–1.2 (0.8 ± 0.1)
Ventral sucker length	0.8–1.0 (0.9 ± 0.03)	1.86–1.5 (1.3 ± 0.03)	1.5–1.9 (1.2 ± 0.08)
Ventral sucker width	0.7–0.9 (0.71 ± 0.04)	0.95–1.2 (1.0 ± 0.03)	0.9–1.8 (1.3 ± 0.11)
Anterior testis length	0.3–0.8 (0.6 ± 0.8)	0.8–1.5 (1.4 ± 0.013)	0.2–0.4 (0.3 ± 0.03)
Anterior testis width	0.2–0.5 (0.4 ± 0.04)	0.9–1.5 (1.2 ± 0.12)	0.4 0–0.9 (0.8 ± 0.08)
Posterior testis length	0.3–0.5 (0.4 ± 0.03)	0.7–1.4 (1.0 ± 0.7)	0.2–0.5 (0.3 ± 0.05)
Posterior testis width	0.3–0.7 (0.4 ± 0.03)	0.7–1.9 (1.0 ± 0.11)	0.2–0.9 (0.6 ± 0.07)
Ovary length	0.2–0.3 (0.18 ± 0.11)	0.4–0.6 (0.5 ± 0.03)	0.2–0.3 (0.2 ± 0.03)
Ovary width	0.12–0.16 (0.14 ± 0.01)	0.2–0.5 (0. 3 ± 0.03)	0.1–0.3 (0.2 ± 0.01)

### Molecular identification

The six PCR products were purified and then bidirectionally sequenced to confirm the identity of the three Clinostomid species. A comparison of these sequences revealed that those six MCs were deeply embedded in the family Clinostomid. These *Clinostomum* spp. isolates were identified as *C. complanatum* (OQ380615.1 and OQ407866.1), *C. phalacrocoracis* (OQ380614.1 and OQ407867.1), and *E. heterostomum* (OQ380616.1 and OQ407868.1), depending on alignment-based sequence analysis.

In this study, the alignment analysis of *the C. complanatum* sequence (OQ380615 and OQ407866) shared the highest similarity score from 99.68 to 99.31% with other *C. complanatum* sequences available in the GenBank database. The BLAST analysis of the current *C. phalacrocoracis* sequence (OQ380614 and OQ407867) revealed the highest similarity of 99.84–99.28% with other *C. phalacrocoracis* sequences available in the GenBank database. On the other hand, the alignment analysis of the current *E. heterostomum* sequence (OQ380616 and OQ407868) had the highest similarity score, 99.52–98.96% similarity with other *E. heterostomum* sequences available in the GenBank database.

**Fig. 6:**
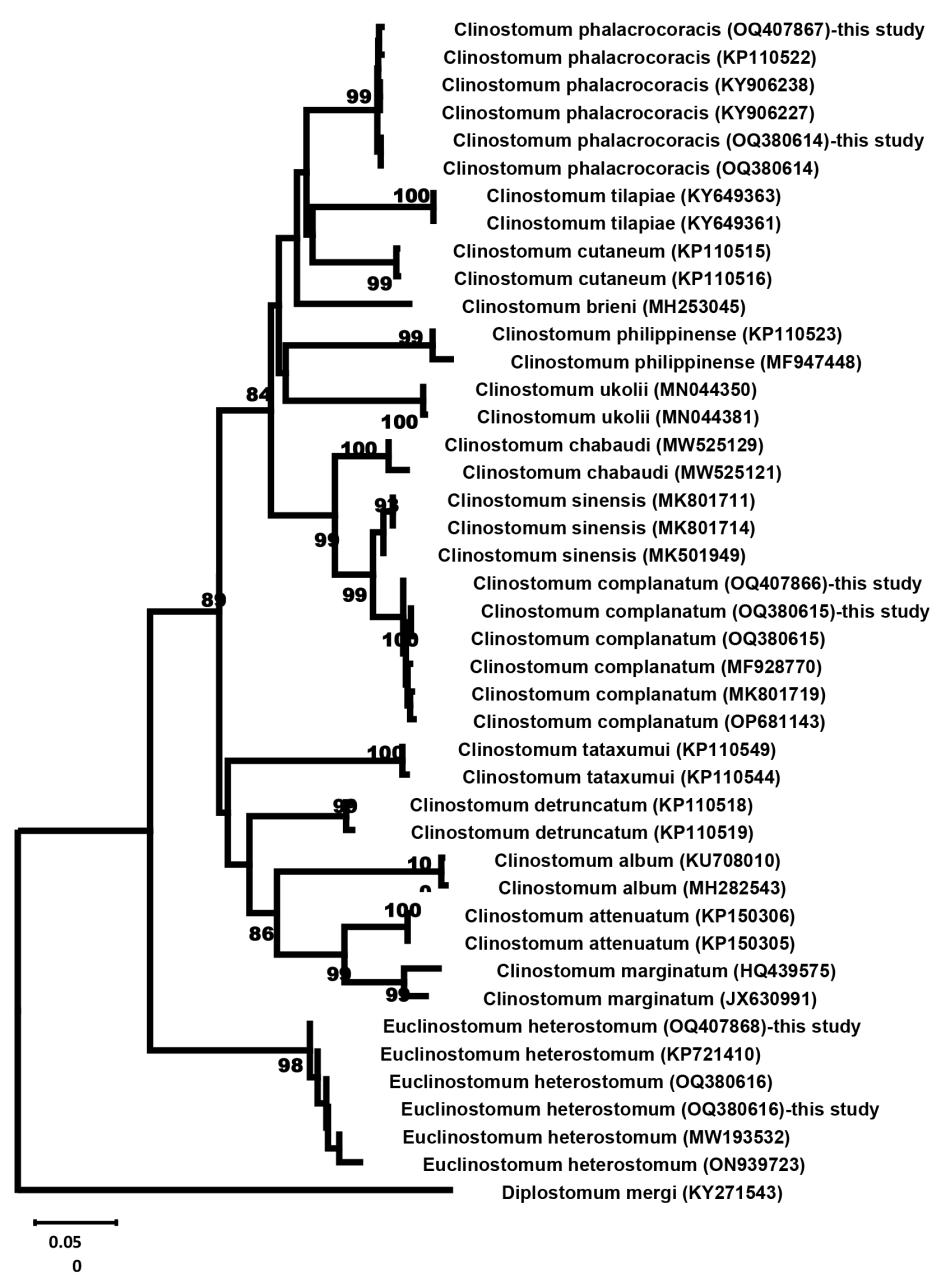
Phylogenetic analysis displaying the comparative alignment of the COI gene sequence of *C. complanatum*,* C. phalacrocoracis*, and *E. heterostomum* with other related clinostomid sequences

The phylogenetic relationships among the current six sequences of Clinostomid against correlated sequences were resolved using the neighbor-joining phylogenetic analysis. Interestingly, the phylogenetic analysis of COI gene sequences revealed two major clades. The first lineage encompassed two subclades. The first subclade was further subdivided into two major branches. The first major branch consists of the current two *C. phalacrocoracis* that were categorized with other *C. phalacrocoracis* with 99% bootstrap value and separated from *C. tilapiae*,* C. brieni*,* C. cutaneum*,* C. philippinense* and *C. ukolii* (Fig. 6). The second major branch contained the current two *C. complanatum* sequences that grouped with other sequences of *C. complanatum* with 100% bootstrap value and separated from *C. chabaudi* and *C. sinensis*. Finally, the second clade formed a monophyletic group of *E. heterostomum* with a 98% bootstrap value distinct from another clade.

### Hematological analysis

The hematological and biochemical analysis results revealed disturbances in the blood parameters, liver, and kidney functions in parasitized tilapia, as shown in (Table 5). Fish infected with MCs showed a decrease in the RBC count and Hb values compared with non-parasitized fish. The WBC count was significantly increased in infected fish. Results also demonstrated an increase in lymphocytes and eosinophils values in diseased fish compared to non-infected fish. On the other hand, neutrophil and monocyte levels decreased in parasitized tilapia in contrast to non-infected fish. Table (5).

**Table 5:** Hematological and biochemical indices of farmed Nile tilapia infected with Clinostomid parasitic infections

Parameter	Non-parasitized	Parasitized	Mann-Whitney U	
			U value	*P*
RBCs (×10^6^/µl)	1.98 ± 0.07	1.36 ± 0.13	0.000	0.050
Hb (g/dl)	7.15 ± 0.08	5.30 ± 0.04	0.000	0.050
WBCs (×10^3^/µl)	4.98 ± 0.06	6.88 ± 0.31	0.000	0.050
Lymphocytes (%)	58.53 ± 1.32	60.12 ± 0.89	1.000	0.127
Neutrophils (%)	35.31 ± 3.52	33.7 ± 5.7	4.000	0.827
Eosinophils (%)	3.24 ± 0.95	4.13 ± 1.08	3.000	0.513
Monocytes (%)	2.92 ± 0.76	2.05 ± 0.57	1.000	0.127
Total protein (mg/ml)	34.68 ± 1.3	29.19 ± 2.02	0.000	0.050
Albumin (mg/ml)	7.36 ± 1.17	4.85 ± 0.86	0.000	0.050
Globulin (mg/ml	27.32 ± 2.01	24.34 ± 1.07	1.000	0.127
Glucose (mg/dl)	71.29 ± 0.81	99.44 ± 0.59	0.000	0.050
Cholesterol (mg/dl)	161.2 ± 1.73	198.7 ± 2.60	0.000	0.050
ALP (Unit/L)	24.52 ± 1.81	39.34 ± 1.20	0.000	0.050
AST (Unit/L)	129.58 ± 1.60	171.55 ± 0.44	0.000	0.050
ALT (Unit/L)	36.08 ± 0.41	45.25 ± 0.61	0.000	0.050
Creatinine (mg/dl)	0.77 ± 0.11	1.57 ± 0.06	0.000	0.050
Uric acid (mg/L)	42.51 ± 0.62	60.16 ± 4.19	0.000	0.050
Lysozyme activity (Unit/ml)	420.00 ± 9.27	369.00 ± 6.55	0.000	0.050

The serum biochemical parameters showed a significant decrease in the total protein, albumen, and globulin values in parasitized tilapia in contrast to non-infected fish. Results also revealed a significant increase in glucose, cholesterol levels, ALP, AST, ALT, Creatinine, and Uric acid values in parasitized fish than in contrast to non-parasitized fish. On the other hand, lysozyme activity significantly decreased in parasitized fish in contrast to non-infected fish. 

### Treatment trials with praziquantel

Treatment of fish with the different PZQ regimens significantly decreased the number of live *Clinostomum* parasites compared to control non-treated fish (Table 6). Exposure of fish to low PZQ concentrations significantly reduced the number of viable metacercariae (highly movement) and was more effective than exposure to high dosage for a short time. The most effective treatment regime was noticed in fish treated twice with 2 mg/ L praziquantel /24 h, Table (6). No mortalities were recorded in all fish groups treated with praziquantel, indicating drug safety. Non-treated fish showed 10% mortality during the trial. Treated fish showed no abnormal clinical manifestations after ending the treatment regimes. Lethargy and excessive mucus secretions were commonly noticed in non-treated fish.

**Table 6:** Efficacy of praziquantel in treating clinstomid infections in tilapia

Praziquantel Dose (mg/L)	Exposure time (hour)	Trials number	The average number of MCs in 10 fish taken at random/ aquarium	Percent of dead MCs
0	-	-	85 ± 6	0%
0.5	24	1	101 ± 9	48%
2	24	1	109 ± 3	60%
3	24	1	79 ± 13	66%
0.5	24	2	121 ± 6	70%
2	24	2	95 ± 3	83%
3	24	2	68 ± 8	77%
4	4	1	92 ± 10	25%
8	4	1	88 ± 5	44%

### Histopathology

**Fig. 7:**
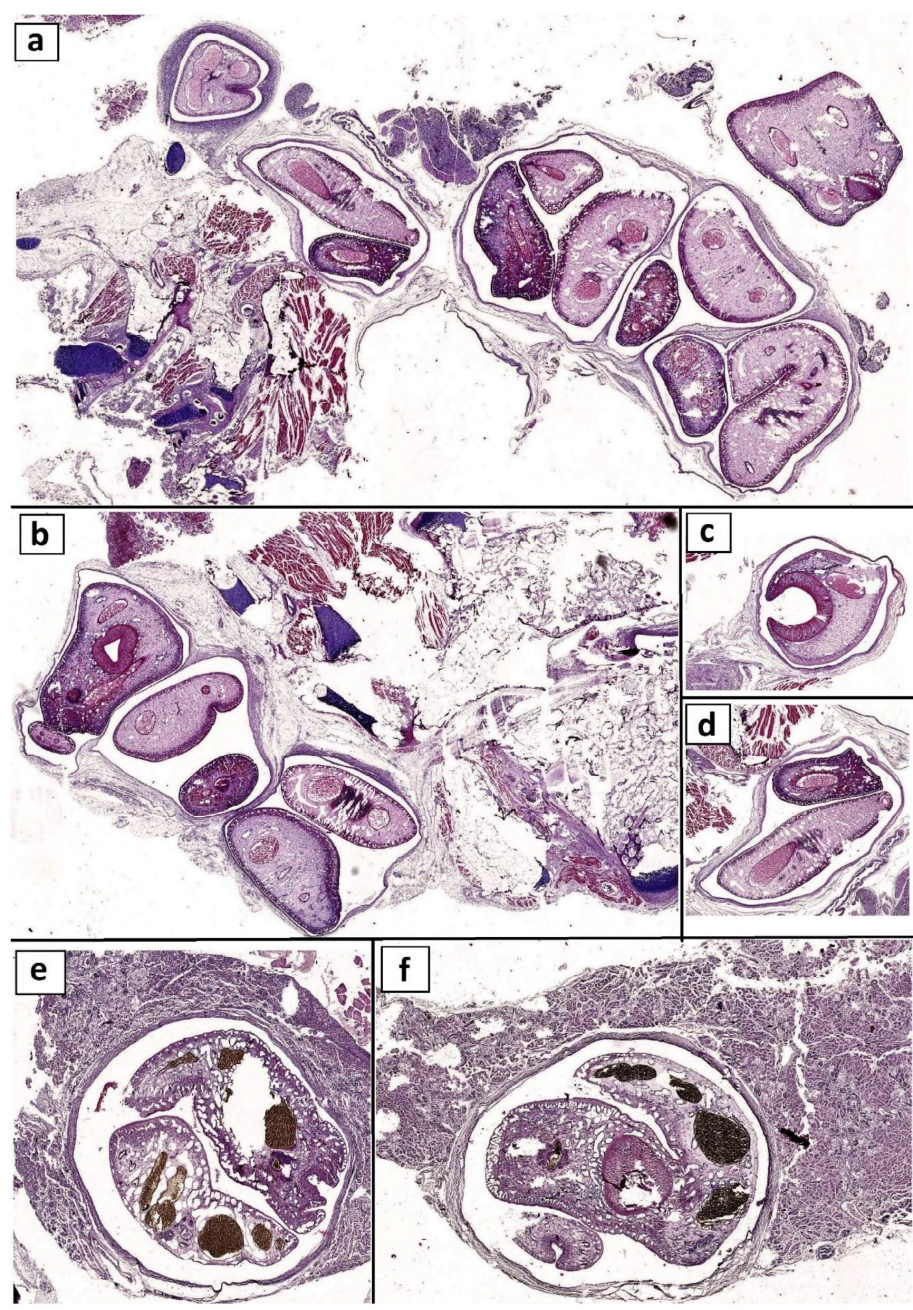
Photomicrograph of buccal cavities and kidney of fish (H&E stain) a-d Sub-gross appearance showing the presence of numerous encapsulated *Clinostomum* parasite embedded in the connective tissue with inflammatory cells infiltration and degeneration of surrounding muscle bundles. e and f showing the effacing of renal parenchyma with parasitic cysts containing *Euclinostomum* parasite

**Fig. 8:**
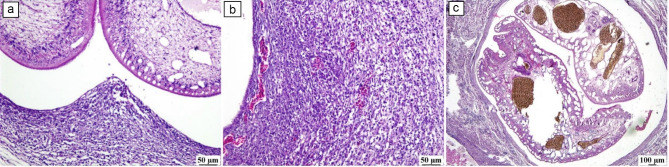
Photomicrograph of fish (H&E stain).(**a-e**) *Clinostomum* capsules parasites with severe inflammatory response. (**f**) *Euclinostomum* parasite replacing the renal tissue with degeneration of the surrounding renal tubules

Examination of the buccal cavities and connective tissue of the affected fishes revealed the presence of numerous *Clinostomum* spp. parasites with pressure atrophy and necrosis of adjacent muscle bundles. Multiple parasitic cysts were noticed embedded in tissues and replaced their typical structures. The parasitic cysts were bounded by thick connective tissue capsules, and severe inflammatory reactions were noticed in the surrounding tissue the parasite was encapsulated with fibrous connective tissue capsules associated with numerous inflammatory cell infiltrations. Numerous EMCs were observed in the base of gills and embedded in the muscle bundles with the absence of minimal inflammatory reaction. (Fig. 7). The kidney revealed the replacement of the renal parenchyma with large parasitic cysts containing *E. heterostomum* trematode species that were encapsulated with fibrous connective tissue capsules accompanied by degenerative changes in the adjacent renal tubules (Fig. 8).

## Discussion

Maintaining adequate water quality measures and good management practices is essential for keeping fish healthy. Long-term exposure of aquatic animals to degraded aquatic environments results in a stress response, rendering them more susceptible to numerous pathogens [32–34]. The occurrence of fish parasitic diseases is closely linked to water sanitary conditions [35]. In this study, unfavorable environmental conditions and poor water quality parameters may have contributed to the appearance of Clinostomid MCs in farmed and wild tilapia, consistent with earlier studies [36]. The tilapia farm exhibited the worst physicochemical water characteristics compared to discharge canals, likely due to high fish stocking density, nutrient loads, and organic matter in affected earthen ponds. In the current study, the dissolved oxygen levels in farm water were below the recommended 5 mg/L, this result consistent with earlier study [37]. In addition, notably, dissolved oxygen availability decreased as water temperature increased [38]. The decrease of dissolved oxygen levels caused by overfeeding, excessive pond fertilization, and phytoplankton overgrowth Unfavorable ammonia concentrations were observed, with the highest values in tilapia fish farms, attributed to over-intensification of aquaculture practices, decomposition of uneaten feed, and poor management [39]. Non-ionized ammonia acts as a significant stressor, influencing fish health and lowering resistance to pathogens [40]. Water analysis also revealed the presence of coliforms, indicating fecal contamination. Livestock and piscivorous birds observed at collection sites can introduce numerous pathogens into the water [41]. Regarding the zoonotic potential of Clinostomid, it is important to note that these parasites pose a risk to human health. Moreover, birds, which are significant vectors of some parasitic diseases affecting fish and humans [42, 43], can transfer parasitized fish over vast distances, spreading infections between fish stocks and between farmed and wild populations [44]. The clinostomid parasites can impair fish’s growth, health, physiology, and productivity [45]. The observed respiratory distress in parasitized tilapia is induced by the presence of multiple MCs stages in the buccal cavity with their destructive suckers, which irritate and disrupt the organ’s function [12, 46]. Through the parasite’s intermediate hosts, pollutants may also have a direct or indirect impact on the free-living phases of the parasite life cycle. As a result, tracking changes in the parasite’s infection values in the host is simpler than utilizing standard techniques to calculate pollution levels [47].

The sequencing and BLAST analysis of the COI gene proved the identity of *C. complanatum*,* C. phalacrocoracis*, and *E. heterostomum* recovered from farmed and wild tilapia. The sequencing of the COI gene serves as a DNA barcoding region that can be used to resolve the taxonomic ambiguity among deeply related digenetic trematodes [28]. In addition, sequencing of the COI region proved better species identity among narrow taxonomic groupings of Platyhelminthes rather than using ITS region [13]. Therefore, the combination of morphological features of Clinostomid and sequencing of the COI gene has been effectively employed to differentiate among closely related *Clinostomum* spp. as previously documented by [8, 12]. In the present study, he recovered encysted worms were observed in the buccal cavity, and kidneys at the base of the fins. Infection sites of Clinostomid species were consistent with earlier studies [16].

In the present result, they find the stressful value of ammonia with high levels (1.58 ± 0.0) and low levels of dissolved oxygen (4.1 ± 0.21) from farm water were detected. This result in agrees [48], who reported heavy metals, acidification, sewage sludge, thermal effluent, pulp-mill effluent, industrial effluent, eutrophication, and unidentified human disturbance. Heavy metals and “unspecified human disturbance” led to a decrease in parasitism, while eutrophication increased it [49], reported some factors can lead to mass fish deaths through the deterioration of host immunity.

The study findings revealed the damaging effects of Clinostomid MCs on tilapia health with analysis of the haematological and biochemical parameters showed physiological disturbances in parasitized fish [29]. In current study, revealed significant increases in WBC count and lymphocytes in infected fish, in agreement with previous studies [50]. The increase of these cells may be a defense mechanism of the fish’s immune system to resist infection [51]. Results also revealed an increase in eosinophils in parasitized fish. The eosinophils are one of the critical immune responses of fish infected by parasitic worms [52]. The study findings also revealed a significant increase in glucose levels in parasitized fish, in agreement with earlier studies on parasitic infections affecting fish [53]. The increase in the ALP, AST, and ALT levels in infected fish is in agreement with the consistent record [54]. These enzymes are commonly elevated in parasitized fish and are related to tissue injury and liver dysfunction [55]. The high creatinine and uric acid values in parasitized fish are related to kidney dysfunction induced by these parasites [33].

Furthermore, Nile tilapia treated with different praziquantel regimens showed a significant reduction in the number of live Clinostomid compared to non-treated fish. The results showed that the prolonged treatment with low PZQ concentrations was more effective in killing the Clinostomid worms than short exposure to high drug dosage. The most effective treatment protocol was observed in fish treated twice with 2 mg/L PZQ / twenty-four hours. The PZQ in the water is absorbed through the gills, and the skin then distributes to the rest of the body tissues [56] and is mainly excreted via the kidneys [57]. Results are similar to previous reports indicating the effectiveness of praziquantel against a broad range of trematodes and cestodes infections in fish [19, 20]. In the current study, the absence of mortality in all the treated groups indicates and indicates the safety of praziquantel administration in tilapia fish, this result, in agreement with the results studied the drug’s pharmacokinetics after a single dip treatment of rockfish with PZQ (100 mg/L) for 4 min [56].

There is no specified residue limit for PZQ in fish sold for human consumption [19]. Elimination of PZQ from the host tissues occurs quickly [56] examined the elimination of PZQ after treatment of rockfish with an oral dose (400 mg/kg) for 3 days. They found no detectable limits of PZQ at 7 days post-treatment. This finding have the way for developing novel treatment in *Clinostomum* worm, targeted strategies to control fish-borne zoonotic trematodes, potentially reducing their impact on public health and aquaculture economies. Furthermore, further study could be contributed to the growing body of evidence supporting the use of nanomaterials for parasite control in aquaculture and other applications [57, 58]. Numerous histopathological alterations were noticed in tissue sections prepared from infected tissues; similar to those in earlier studies [7, 46] MCs were embedded in tissues and replaced their typical structures. The parasitic cysts were surrounded by dense connective tissue capsules, and severe inflammatory reactions were noticed in the surrounding tissue. Lymphocytic infiltration, edema, and necrotic and degenerative changes were noticed in infected tissues in agreement with previous reports [12, 17].

## Conclusion

In the current study, Clinostomid infections; *C. complanatum*, *C. phlacrocorasis* and *Euclinostoum heterostomum* are dangerous pathogens in farmed and wild Nile tilapia. The presence of these parasites reduces fish marketability, and some are zoonotic trematodes such as *C. complanatum*. Clinostomid infections increase with inadequate water quality measures and bad management practices. The abundance of snails and predatory birds can increase the occurrence of these parasitic infections in fish. The abundance of snails and predatory birds can increase the occurrence of these parasitic infections in fish. The investigated geographical areas are characterized by freshwater ecosystems heavily impacted by bird activity, leading to a prevalence of fish parasites due to factors such as pollution and environmental degradation. This study demonstrates that Clinostomid infections induce significant hematological, biochemical, and histopathological alterations in fish. Controlling these infections is crucial for improving fish health, maximizing profits, and ensuring the sustainability of the fish industry. Praziquantel has proven to be an effective and safe treatment for *Clinostomum* infections in farmed Nile tilapia. Waterborne exposure to praziquantel (2 mg /L) for twenty-four hours provides a safe and efficient therapy for controlling these infections in Nile tilapia. Increased awareness among fish farmers, good management practices, and proper management for predators and snails are essential measures to control Clinostomid infection.

## Data Availability

Availability of Data and Materials All the authors declare that all the data supporting the results reported in our article were included in this article. The datasets generated or analyzed during the current study are available in the GENEBANK repository, with accession numbers *C. complanatum* (OQ380615.1 and OQ407866.1), C. phlacrocorasis (OQ380614.1 and OQ407867.1) and *Euclinostoum heterostomum* (OQ380616.1 and OQ407868.1), respectively.-https://www.ncbi.nlm.nih.gov/nuccore/OQ380614.1-https://www.ncbi.nlm.nih.gov/nuccore/OQ380615.1-https://www.ncbi.nlm.nih.gov/nuccore/OQ380616.1-https://www.ncbi.nlm.nih.gov/nuccore/OQ407866.1-https://www.ncbi.nlm.nih.gov/nuccore/OQ407867.1-https://www.ncbi.nlm.nih.gov/nuccore/OQ407868.1.
